# Influence of the Intensity, Components, and Spreading of the Deqi Sensation on the Analgesic Effect of SP6 Needling in Primary Dysmenorrhea Patients: A Secondary Analysis of a Randomised Controlled Trial

**DOI:** 10.1155/2019/6462576

**Published:** 2019-05-16

**Authors:** Ni-juan Hu, Yu-qi Liu, Min-yi Zhao, Pei Wang, Gui-wen Wu, Shang-qing Hu, Jun-jun Sun, Ya-feng Wang, Zhuang Zhang, Liang-xiao Ma, Jiang Zhu

**Affiliations:** ^1^School of Acupuncture, Moxibustion and Tuina, Beijing University of Chinese Medicine, Beijing 100029, China; ^2^Institute of Basic Research in Clinical Medicine, China Academy of Chinese Medical Sciences, Beijing 100700, China; ^3^Ennova Health Science and Technology Co., Ltd., ENN Group, Hebei 065001, China; ^4^Beijing Luhe Hospital Affiliated to Capital Medical University, Beijing 101149, China; ^5^The First People's Hospital of Changzhou, Jiangsu 213003, China; ^6^The Second Affiliated Hospital of Xi'an Jiao Tong University, Shaanxi 710004, China; ^7^The Key Unit of State Administration of Traditional Chines Medicine, Evaluation of Characteristic Acupuncture Therapy, Beijing 100029, China

## Abstract

Although deqi, the phenomenon whereby excitation of Qi in the meridians occurs with needling, is critical to the practice of acupuncture and its efficacy, it is poorly understood. So we investigate the influence of the deqi sensation on the analgesic effects of acupuncture in patients who were enrolled in a randomised controlled trial for the treatment of patients with primary dysmenorrhea, a painful and common condition, and* cold and dampness stagnation*. Two groups were assessed: a deqi group (undergoing deep needling with thick needles and manipulation, n=17) and a non-deqi group (undergoing shallow needling with thin needles and no manipulation, n=51). The Sanyinjiao (SP6) was needled for 30 min in both groups. Pain scores at baseline, upon needle removal, and at 10, 20, and 30 min after needle removal were evaluated by the Visual Analogue Scale for pain. The deqi sensation was evaluated by the Acupuncture Deqi Clinical Assessment Scale. Patients who experienced a genuine deqi sensation (n=39) were selected for further analysis. Compared with patients in the non-deqi group who experienced deqi (n=25), patients who self-reported deqi in the deqi group (n=14) felt a stronger deqi sensation, experienced soreness and fullness more frequently, felt a greater intensity of soreness, fullness, electric sensation, spreading, and radiating, and experienced larger spreading distances. In those who experienced the deqi sensation in the deqi group, the intensity of the sensation, as well as their degree of soreness and fullness, was negatively correlated with pain reduction. In patients who experienced the deqi sensation in the non-deqi group, deqi intensity was positively correlated with pain reduction, while soreness was negatively correlated with pain reduction. The distance of spreading was not correlated with pain reduction in either group. We found, in SP6 needling of patients with primary dysmenorrhea with* cold and dampness stagnation*, that a moderate deqi response predicted a prolonged analgesic effect better than a strong deqi response.

## 1. Introduction

Dysmenorrhea is a common pelvic pain disorder [1]. Primary dysmenorrhea (PD) is a gynaecological disease with no identifiable pathology which has an adverse impact on the lives of many young women, such as school or work absenteeism [2, 3]. Drugs, including nonsteroidal anti-inflammatory drugs (NSAIDs), can reduce the pain of PD by inhibiting the production and release of prostaglandins. However, side effects, such as headaches, dizziness, and drowsiness, can occur with long-term NSAID use [4]. As such, alternative therapies, and especially acupuncture, have become an important alternative to drug-based PD treatment.

Previous research has found that acupuncture can achieve significant pain reduction and relief of primary dysmenorrhea when compared with no treatment, pharmacological treatment, or treatment with herbal medicine alone [5, 6]. Studies have further suggested that individuals who underwent acupuncture treatment experienced lower levels of pain than those who received sham treatments [7–9]. Previous findings suggest that acupuncture controls pain via stimulating the neural receptors and pathways that block pain impulses by interacting with mediators such as endogenous serotonin and endorphins [10]. Maciocia believed that applying stimulation at the spleen meridian can invigorate blood supply and reduce pain [11]. Sanyinjiao (SP6), or the three-channel connection (between the spleen meridian, liver meridian, and kidney meridian), is one of the most commonly used points to treat PD via both acupuncture and acupressure [12, 13]. Even when needling occurs only at SP6 in patients with PD, the resultant analgesic effect is considerable [9, 14, 15]. This is especially evident among patients who also have the traditional Chinese medicine (TCM) condition referred to as* cold and dampness stagnation* [16], a pattern/syndrome marked by cold and pain in the lower abdomen, dysmenorrhea or delayed periods with dark menstrual discharge, white tongue coating, and sunken tight pulse [17]. From the viewpoint of TCM, women suffering from cold or drinking much cold during menstrual periods would develop a cold and dampness stagnation in the uterus with blood stasis and poor circulation which result in pain in the lower abdomen. In the acupuncture treatment of PD, regulation of the uterus with SP6 acupoint is mainly based on the immediate improvement of uterine arterial blood flow [18]. Needling of SP6 in PD patients also activates the cortical and subcortical limbic systems and pain-related areas in the cerebellum [19].

While acupuncture is broadly effective for pain relief, there is a long-held view in the field that needling is effective only when deqi is obtained (*Lingshu Jing *or* Spiritual Pivot, chapter 1*) [20]. This contention was held by practitioners throughout multiple ancient Chinese dynasties and continues to influence the modern practice of clinical acupuncture [21–23]. The term “deqi” is derived from* Huang Di Nei Jing*, translated into “the obtaining of Qi or the arrival of Qi.”

Contemporary researchers have increasingly paid attention to the role of deqi in acupuncture treatment. Deqi is often perceived by patients as a unique physical response (patients usually feel soreness, numbness, and fullness sensation in the area surrounding the acupuncture needle) and by practitioners as needle grasp (acupuncturists feel increased resistance to continued needle manipulation) [21]. As it is difficult to pick up on the perception of the acupuncturist, modern clinical studies of deqi generally focus on the patient sensation. Understanding the relationship between the experience of deqi and the efficacy of acupuncture is one of the most important aspects of deqi research. However, the current literature does not support a clear conclusion on this issue [24, 25]. A study treated osteoarthritis via real and sham needles and found that patients who experienced deqi achieved better treatment effects than those who did not [26]. However, another two clinical studies found no significant difference in pain relief between those who felt deqi and those who did not in patients with osteoarthritis [27, 28]. Additional studies should be conducted to elucidate the influence of deqi on the therapeutic effect of acupuncture.

Our team previously performed a prospective multicentre randomised controlled trial to elucidate the relationship between deqi and the effects of acupuncture on pain. The results of this trial revealed that deqi predicts improved analgesic effects in patients with PD [29]. In the current study, we further analysed these data by focusing on how the intensity, components, and spreading of the deqi sensation relate to the analgesic effects of SP6 needling in patients with PD and* cold and dampness stagnation*. We also investigated the effects of stimulus intensity on deqi sensation intensity, components, and spreading.

## 2. Methods

### 2.1. Study Design

This study utilized a randomised, assessor and patient-blinded, parallel-group trial design. The ratio of patients in the deqi group to those in the non-deqi group was 1:3. We followed the Consolidated Standards of Reporting Trials and the Revised Standards for Reporting Interventions in Clinical Trials of Acupuncture in designing this study and in reporting our findings [30, 31]. This trial was approved by the Ethics Committee of Beijing University of Chinese Medicine (BUCM) (no. 2014BZYLL0302) and registered at the Chinese Clinical Trial Registry (no. ChiCTR-IPR-14005361) on October 21, 2014.

### 2.2. Patients

Participants came mainly from four universities and were recruited using poster advertisements. Posters briefly stated the inclusion and exclusion criteria, participation incentive (opportunity to receive free acupuncture treatment), and the appropriate contact information for enrolment requests.

Participant inclusion criteria were as follows: (1) 18 to 30 years of age; (2) nulliparity; (3) diagnosis with PD according to Primary Dysmenorrhea Consensus Guidelines [32] and with* cold and dampness stagnation* pattern based on the revised Chinese national guideline [33]; (4) duration of PD (self-reported pain) from 6 months to 15 years; and (5) average pain intensity of 40 or greater on a 100 mm Visual Analogue Scale (VAS) for at least three consecutive menstrual cycles.

Participant exclusion criteria were as follows: (1) secondary dysmenorrhea (e.g., caused by endometriosis or adenomyosis); (2) irregular/infrequent menstrual cycles (outside a typical range of 21 to 35 days); (3) pregnancy; (4) prior knowledge of acupuncture; (5) intake of analgesic medication in the 24-hour period prior to needling intervention; (6) asthma, psychological diseases or life-threatening conditions (e.g., disorders of the cardiovascular, liver, kidney or hematopoietic systems); and (7) potential for poor treatment compliance, such as tendency to fear or resist acupuncture needling.

The primary symptom diagnostic criteria used to define* cold and dampness stagnation* were as follows: (1) sensation of cold and pain in the lower abdominal before or during the menstrual period, alleviated by warmth; (2) delayed menorrhea and hypomenorrhea; (3) dark, clotted menses; or (4) black menses.* Secondary symptoms* included (1) cold intolerance; (2) lack of warmth in the extremities; (3) profuse vaginal discharge; (4) white and greasy tongue coating; (5) weak pulse; or (6) sunken and tight pulse (a deeply located pulse which can only be felt when pressing hard and the pulse feeling like a tightly stretched cord). Each primary symptom was scored as 2 points while secondary symptoms were scored as 1 point. Primary and secondary symptom scores were added together to give a total score.* Cold and dampness stagnation* was confirmed if the total score was greater than or equal to 6.

### 2.3. Study Procedures

Candidates with an expressed interest in participation were initially assessed via telephone. After providing written consent, candidates participated in a comprehensive face-to-face interview with a researcher. Eligible candidates were informed of the procedures and potential risks of the study and a full medical and gynaecological history was collected by two researchers. Further examination and ultrasonography was performed on participants who passed the preliminary evaluation and provided signed consent. Every participant who met all inclusion criteria was enrolled.

### 2.4. Intervention

Treatments were performed in the laboratory of the School of Acupuncture, Moxibustion and Tuina at the BUCM. A registered Chinese medical practitioner with five years of experience in providing acupuncture was responsible for all acupuncture treatments. The trial was also periodically supervised by an experienced acupuncturist and a doctoral candidate to ensure the trial quality.

During menstrual dysmenorrhea, patients with abdominal pain of 40mm or greater underwent acupuncture treatment. All patients underwent needling of the bilateral SP6 for 30 min while being in a supine position. SP6 is located on the tibial aspect of the leg, posterior to the medial border of the tibia and 3 B-*cun* (proportional bone* cun*), above the prominence of the medial malleolus [34]. Sterile disposable stainless steel acupuncture needles with guide tubes were used (Zhongyan Taihe, Wuxi Jiajian Medical Instrument Co. Ltd., Jiangsu, China).

In the deqi group, 0.3 × 40 mm stainless steel needles were inserted into SP6. The depth of insertion was 1.0–1.2* cun*; needles were then manipulated by lifting-thrusting and twirling (180° in both directions, once per second) for 30 s. This manipulation was repeated twice every 10 min for 30 s each time. The aim of this treatment was to achieve deqi as frequently and significantly as possible.

In the non-deqi group, 0.18 × 13 mm stainless steel needles were inserted into SP6 to a depth of 0.1-0.2* cun* and were not manipulated. The aim of this treatment was to avoid achieving deqi.

### 2.5. Measurements

The primary outcome assessed in this trial was abdominal pain, as measured by the Visual Analogue Scale for pain (VAS-P). This was assessed at baseline (VAS-P_0_), at needle removal (VAS-P_30_), and then at 10 (VAS-P_40_), 20 (VAS-P_50_), and 30 (VAS-P_60_) min after needle removal. The secondary outcome was the experience of the deqi sensation, as measured by the Acupuncture Deqi Clinical Assessment Scale (ADCAS). This scale has shown good reliability and validity in a past study by our group (manuscript in preparation). Per the ADCAS, the intensity of needle sensation was evaluated using a five-level scale (0: not at all; 1: mild; 2: moderate; 3: strong; and 4: unbearably sharp pain). Next, twelve components of the needle sensation (dull pain, warmth, coldness, soreness, numbness, fullness/distention, heaviness, electric sensation, formication, throbbing, spreading, and radiating) were scored using four-level (0: not at all; 1: mild; 2: moderate; 3: strong) scales. In addition, patients were asked to describe the sensation they experienced in their own words if they felt that the twelve descriptors did not accurately or completely express their experience. The ADCAS also contains a subscale to evaluate the spreading of the deqi sensation. Using the ADCAS, we recorded scores of the needle sensation intensity, the twelve components of sensation, and the bilateral spreading of the deqi sensation at SP6.

Often, the purpose of acupuncture is inconsistent with the sensory experience elicited by it; despite an acupuncturist wanting to induce the deqi sensation, the patient may not experience anything, deqi included, as a result of needling. Therefore, to judge the efficacy of acupuncture treatment, it is necessary to assess the patient's response in a clinical setting. Some experts contend that the intensity of the overall patient experience of needling sensation may be a gross marker of the adequacy of acupuncture treatment, more broadly [35].

In the present study, we used the overall intensity of the needle sensation to judge whether a patient experienced deqi. Overall needle sensation intensity was calculated as the sum of the intensity scores of the bilateral needling sensation at SP6 and ranged from 0 to 8. In accordance with the general view of clinical acupuncturists, sharp pain (scores of 4 on the ADCAS) should be avoided as these are not conducive to deqi [36]. Here, five conditions were rated as non-deqi, namely, 0+0 (no sensation at all), 0+1 (a weak unilateral sensation), 0+4 (no sensation on one side and sharp pain on the other side), 4+4 (bilateral sharp pain), and 1+4 (a weak sensation on one side and sharp pain on the other side). With the exception of these five conditions, all other states (0+2, 0+3, 1+1, 1+2, 1+3, 2+2, 2+3, 2+4, 3+3, 3+4) were judged as deqi [37]. Pain sensation caused by the penetration of the needle into the skin is not confined to deqi. Using these parameters, all patients were classified according to their actual experience of deqi. The incidence of all adverse events was also recorded.

### 2.6. Sample Size

Our sample size was based on a calculation for sample size estimation in studies with a repeated measurements design [38]. According to the recommendations of the Initiative on Methods, Measurement, and Pain Assessment in Clinical Trials, the minimal clinically effective value for change in abdominal pain is 10 mm in dysmenorrhea patients [39]. In our previous study, the average abdominal VAS-P value before treatment was 15.934 [29]. Thus, our initial sample size was determined to be 14 for each group, with 70% power and *α*=0.05. Further, in our prior study, the sizes of the two groups were equal and patients in the deqi group all experienced actual deqi, while only approximately 1/3 of patients in the non-deqi group failed to experience deqi. Thus, our comparison of genuine deqi or no deqi was limited by a great disparity in the final number of cases in each group. To compare deqi and non-deqi using a population size as similar as possible in the present study, the size of the non-deqi group thus needed to be three times that of the deqi group. As such, the ratio of deqi group to non-deqi group participants in the present trial was roughly 1:3. Accounting for an estimated dropout rate of 20%, the final sample sizes were 17 and 51 in the deqi and non-deqi groups, respectively.

### 2.7. Randomisation and Blinding

Patients were randomised using a 1:3 allocation ratio (deqi: non-deqi group patients), conducted by an independent administrator using a computer-generated list of numbers. Allocation codes and procedural information were sealed in sequentially numbered opaque envelopes and kept by an independent administrator. Randomisation was carried out via a centralised telephone procedure. Five to ten minutes before treatment, the administrator informed the acupuncturist of the participant's assignment by telephone. Only the acupuncturist knew the group allocation and treatment assignment of each patient. The patients and the recorder who assessed all study outcomes were unaware of the treatment assignment and were blinded to the procedure; a screen was placed between the recorder and the acupuncturist to ensure that the recorder and patients could not see the process. The acupuncturist wore earplugs to avoid being affected by vocal self-reporting from patients during needling stimulation.

### 2.8. Data Analysis

The present is a secondary analysis of patients who experienced the deqi sensation in a prior study. Baseline characteristics were described by arithmetic means and standard errors (SEs). Means of independent samples were compared by t-tests or non-parametric rank-sum tests, as appropriate. A Chi-square test was used to compare rates in the two samples. Pearson's or Spearman's correlation coefficients were used for correlation analyses. P values that were less than or equal to 0.05 were considered to be statistically significant.

## 3. Results

### 3.1. Baseline Characteristics

**Figure 1:**
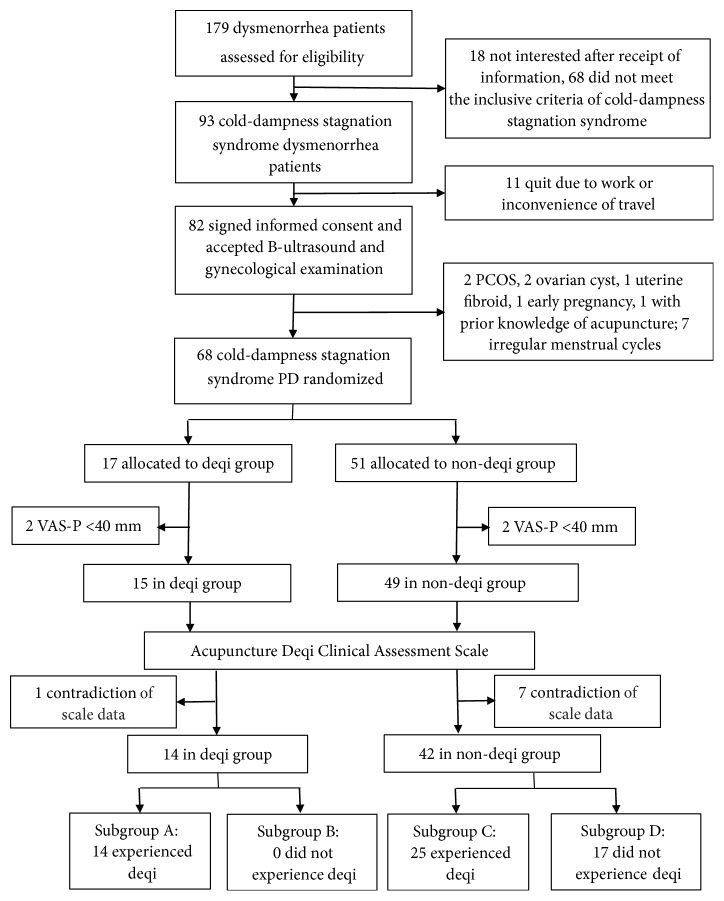
Experimental design. PCOS: Polycystic ovary syndrome; PD: Primary dysmenorrhea; VAS-P: VAS for pain.

From September 2014 to May 2015, 179 candidates applied to participate in our study, of whom 18 dropped out and 68 did not meet inclusion criteria. Among the remaining 93 participants, 11 later dropped out due to school or work commitments. A final 82 participants provided informed consent and participated in B-ultrasound and gynaecological examinations. Of these, six did not meet PD diagnostic criteria and further eight were excluded for menstrual cycle disorder and unknown background of acupuncture. Of the remaining 68 enrolled participants, four were excluded as their VAS-P values prior to treatment were less than 40 mm (Figure 1). Complete baseline information was recorded for 64 patients (deqi group n=15, non-deqi group n=49).

The mean age of patients was 21.52 ± 0.36 years and the average age of dysmenorrhea onset was 15.97 ± 0.30 years old. Participants had an average length of dysmenorrhea history of 67.70 ± 5.36 months and reported an average level of pain on the first day of menstruation of 54.41 ± 1.29 mm. Participant age, PD onset age, period characteristics, menstrual cycle length, disease duration, and VAS-P scores before treatment did not differ significantly between the two groups (Table 1).

### 3.2. Deqi Sensation and Its Correlation with Pain Reduction

According to the ADCAS, one patient in the deqi group and seven patients in the non-deqi group exhibited an inverse relationship between their experience of deqi and their self-reported needling experience. These patients were excluded from secondary analyses (Figure 1). The remaining 14 patients in the deqi group all self-reported an experience of deqi (subgroup A), leaving none who did not report deqi (subgroup B). Of the 42 patients in the non-deqi group, 25 (59.5%) self-reported an experience of deqi (subgroup C). Of these, only 17 patients were considered not to have experienced the deqi sensation (subgroup D). Overall, 39 patients experienced the deqi sensation and 17 patients did not. Subsequent analyses focus only on the 39 patients who experienced the deqi sensation.  Table 2 shows changes over the progression of treatment in pain experienced in subgroups A, C, and both combined.

**Figure 2:**
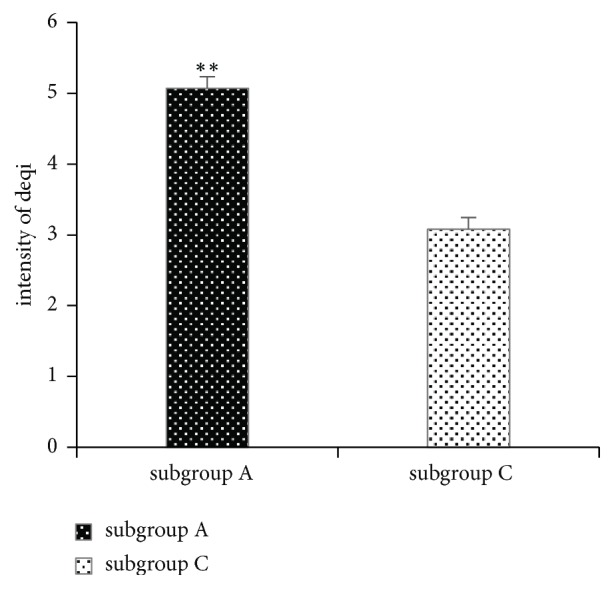
Intensity of deqi in subgroup A and subgroup C. Subgroup A: patients in the deqi group who in fact experienced deqi. Subgroup C: patients in the non-deqi group who experienced deqi. Mann-Whitney U test, with *∗∗* indicating p < 0.01.

In subgroup A, the mean intensity of the reported deqi sensation was 5.07; in subgroup C it was 3.08. The overall intensity of the deqi sensation in subgroup A was significantly greater than that in subgroup C (1.99, 95% CI 1.49 to 2.50; p ≤ 0.001) (Figure 2). Subgroup-wise correlations between the intensity of the deqi sensation and the effects of treatment are shown in Table 3. In subgroup A, there was a negative correlation between the intensity of the deqi sensation and pain reduction from baseline until needle removal (r = -0.554, p = 0.04). In subgroup C, there was a positive correlation between the intensity of the deqi sensation and pain reduction from baseline until 20 min after needle removal (r = 0.422, p = 0.036). There was no correlation between the intensity of the deqi sensation and the effects of acupuncture treatment in subgroups A and C combined (A+C).

**Figure 3:**
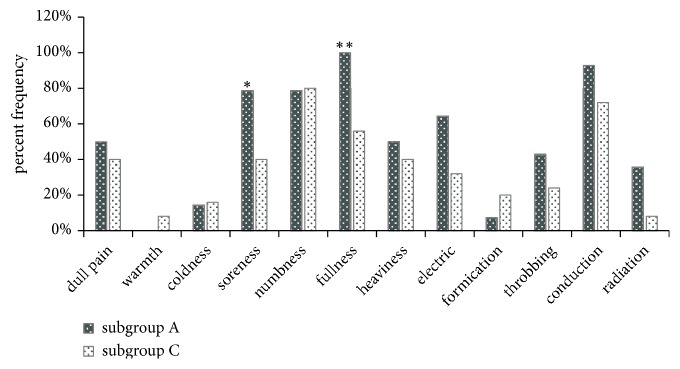
The frequency of different sensations in subgroups A and C. In subgroup A, fullness was the most frequent sensation, followed by spreading, soreness, numbness, and electric sensation. Numbness was the most common sensation in subgroup C, followed by conduction and fullness. Fisher's exact test, with *∗* indicating p < 0.05 and *∗∗* p < 0.01. Subgroup A: patients in the deqi group who in fact experienced deqi. Subgroup C: patients in the non-deqi group who experienced deqi.

**Figure 4:**
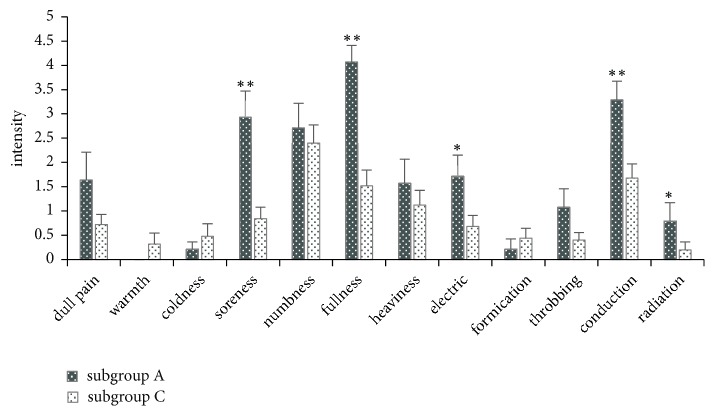
The intensity of different sensations in subgroups A and C. In subgroup A, fullness was the strongest sensation, followed by spreading, soreness, numbness, and electric sensation. Numbness was the strongest sensation in subgroup C, followed by conduction and fullness. Mann-Whitney U test, with *∗* indicating p < 0.05 and *∗∗* p < 0.01. Subgroup A: patients in the deqi group who in fact experienced deqi. Subgroup C: patients in the non-deqi group who experienced deqi.

In subgroup A, the descriptive components with a frequency greater than 50% and an intensity greater than 1.5 were soreness, numbness, fullness, electric sensation, and spreading. Subgroup C self-reported numbness, fullness, and spreading. Among the twelve sensation components, only the frequencies of soreness (p = 0.043) and fullness (p = 0.003) were significantly different between subgroups A and C (Figure 3). However, the intensities of soreness (2.09, 95% CI 0.85 to 3.32; p = 0.001), fullness (2.55, 95% CI 1.6 to 3.5; p ≤ 0.001), electric sensation (1.03, 95% CI 0.13 to 1.94; p = 0.032), spreading (1.61, 95% CI 0.63 to 2.58; p = 0.002), and radiating (0.59, 95% CI -0.29 to 1.46; p = 0.034) were significantly different between these two subgroups (Figure 4). A single patient in subgroup C opted to further describe a sensation of “stiffness” to convey her treatment experience.

**Figure 5:**
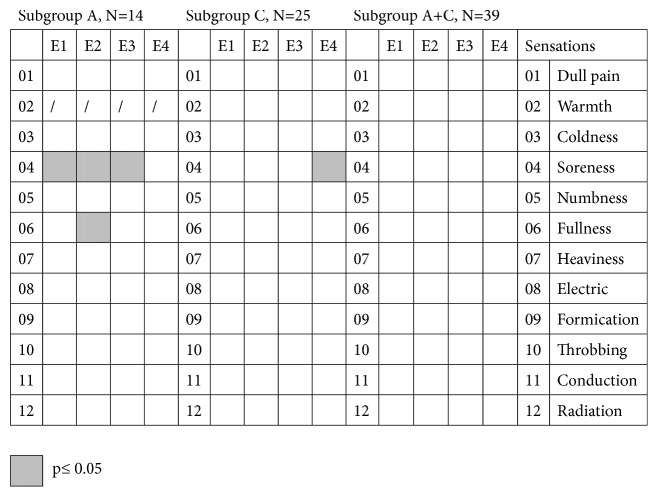
Correlation between intensities of the components of the deqi sensation and their effects on pain. Subgroup A: patients in the deqi group who in fact experienced deqi. Subgroup C: patients in the non-deqi group who experienced deqi. Subgroup A+C: patients who experienced deqi in groups A and C, combined. E1 = VAS-P_0_  − VAS-P_30_, E2 = VAS-P_0_  − VAS-P_40_, E3 = VAS-P_0_  − VAS-P_50_, E4 = VAS-P_0_  − VAS-P_60_. VAS-P_0_-_60_: VAS for pain before treatment, at needle removal, and 10, 20, and 30 min after needle removal (indicated by subscript).

In subgroup A, negative correlations were found between soreness and pain reduction at all timepoints except VAS-P_0_  − VAS-P_60_ (soreness vs. VAS-P_0_  − VAS-P_30_: r = -0.581, p = 0.029; soreness vs. VAS-P_0_  − VAS-P_40_: r = -0.652, p = 0.011; soreness vs. VAS-P_0_  − VAS-P_50_: r = -0.561, p = 0.037) (Figure 5). There was also a negative correlation between fullness and pain reduction from baseline to 10 min after needle removal (r = -0.541, p = 0.046). In subgroup C, there was a negative correlation between soreness and pain reduction from baseline to 30 min after needle removal (r = -0.469, p = 0.018). There were no correlations between the components of the deqi sensation and the effects of acupuncture treatment in subgroup A+C.

In subgroup A, 92.9% of patients self-reported a spreading distance of 3.29 ± 0.41, while in subgroup C, 72% of patients self-reported a spreading distance of 1.94 ± 0.37. There was no significant difference in the frequency of the experience of spreading between subgroups A and C (p = 0.218). However, the distance of spreading in subgroup A was farther than that in subgroup C (1.35, 95% CI 0.17 to 2.52; p = 0.019). There was no correlation between the distance of deqi spreading and the effects of acupuncture treatment in either group.

### 3.3. Safety of Acupuncture

No adverse effects were reported.

## 4. Discussion

The present study explored the influence of deqi sensation intensity, sensory components, and spreading on the pain-ameliorating effects of acupuncture treatment. The premise for this investigation was that deqi improves pain control in acupuncture care, as demonstrated in prior work by our group [40]. To examine this, we maximised differences between the deqi and non-deqi groups examined here by employing different filiform needles (diameter and length), varying needling depth, and using or not using manipulation, respectively. For improved between-group comparisons, we also used the ADCAS to evaluate patients' perceived experience of the deqi sensation and included a subset of patients in whom the deqi sensation was actually perceived. Using this robust approach, we found that the intensity of deqi sensation and certain components of it influenced the analgesic effects of acupuncture treatment in PD patients with concomitant* cold and dampness stagnation*.

### 4.1. The Influence of Deqi on Treatment Effects

Notably, the intensity of the deqi sensation was differentially related to pain relief in the present study. In subgroup A, a higher intensity of the deqi sensation elicited negative pain relief upon needle removal. However, in subgroup C, a higher intensity of the deqi sensation elicited pain relief from baseline to 20 min after needle removal. Based on our previous work [29, 40], we concluded that, while deqi is more effective than non-deqi, stronger deqi does not necessarily result in better outcomes.

In addition, we found that soreness was negatively correlated with pain relief in the present study. This correlation was identified from baseline to 20 min after needle removal (VAS-P_0_  − VAS-P_30_, VAS-P_0_  − VAS-P_40_, VAS-P_0_  − VAS-P_50_,) in subgroup A, but only at 30 min after needle removal in subgroup C (VAS-P_0_  − VAS-P_60_). Fullness was also negatively associated with pain relief from baseline to 10 min after needle removal (VAS-P_0_  − VAS-P_40_). Based on these results, we conclude that, although achieving deqi can predict better treatment effects than non-deqi, an increased intensity of certain components of deqi does not necessarily result in better outcomes. Similar findings have also been reported by Lee et al., who found that a greater improvement in several pain and sensory detection thresholds was associated with weaker acupuncture sensation intensities. The authors further found that heaviness was negatively correlated with changes in the vibration detection threshold and that fullness and throbbing were negatively correlated with changes in the warm detection threshold [41]. Concerning the results from the present study, it seems that a moderate sensory response aroused by acupuncture needling would be propitious for pain relief.

In the present study, we did not find any correlation between spreading and the analgesic effect of acupuncture treatment. In contrast, patients who experienced deqi sensation spreading to the lower limbs or feet in treatment for lumbar intervertebral disc prolapse achieved better therapeutic effects than those who only experienced deqi locally [42]. The cause of this disparity may be that in the present study, we did not apply any special manipulative methods to facilitate directional transmission of deqi to the location of pathology.

The effectiveness and safety of acupuncture for the treatment of PD have been confirmed previously, with acupressure at SP6 being effective for the alleviation of PD symptoms by temperature elevation in the Qugu (CV12) pathway [43]. Yang et al. reported that thermogenic action along the spleen meridian was manifested with simultaneous increases in skin temperature at the right SP6 and Xuehai (SP10) 5-10 min after SP6 needling [44]. This elevation in temperature may be related to an increase in blood flow induced by deqi [45, 46]. Several studies have suggested that deqi involves extensive deactivation of blood oxygen-level dependent signals in multiple regions of the brain, such as mesial prefrontal cortex, medial temporal lobe, middle temporal gyrus, fusiform gyrus, and lingual gyrus [47–50]. Specifically, these regions are related to the limbic-paralimbic-neocortical network which has been implicated by multiple studies as potentially involved in acupuncture analgesia via serotonin and dopamine release [51].

In our prior work, while an alleviation of pain occurred in patients who did not self-report deqi, this effect was greater in those who did [40]. Notably, acupuncturists treating the non-deqi group in our prior trial employed the same acupoint as that used in treating the deqi group, and non-deqi patients experienced invasive needling rather than non-invasive placebo acupuncture (e.g., “non-point” acupuncture). Thus, patients in the non-deqi group here also experienced stimulation of the skin and superficial fascia at the disease-related acupoint, though this stimulation was below the patient's sensory threshold.

Interestingly, wrist-ankle acupuncture, a needling method via inserting the needle through the skin shallowly in the subcutaneous tissue level in the wrist or ankle without manipulation and needle sensation, exerts similar analgesic effect to traditional acupuncture [52]. Xiang et al. found that this shallow and no sensory inputting needling method induced the low-frequency BOLD signal oscillation response in the left insular in patients with chronic low back pain [53]. Concerning the results of the current study, it seems that analgesic effects achieved by invasive needling at an acupoint without deqi may be correlated with some kinds of activities among some structures in the brain. Further studies could pay attention to the mechanism about this phenomenon.

### 4.2. The Influence of Needling Intervention on Deqi

Deqi is a form of bodily meridian reaction that occurs as a response to needling intervention in acupuncture. The deqi sensations involve a wide spectrum of afferent nerves, ranging from coarse myelinated A*β* fibres to fine unmyelinated C fibres [54–57]. Hui et al. found that aching, soreness, dull pain, and warmth were all associated with slower conduction in A*δ* and C fibres and that pressure invokes A*γ* and A*δ* nerve fibre responses while numbness and tingling involve A*β* fibres [58]. That is, the deqi sensations perceived by patients are produced by needling different afferent nerve fibres.

Despite commonalities in neuroanatomy, individuals may have different responses to stimuli of different intensities. In the present study, patients who received deep needling with thick needles and manipulation experienced stronger deqi sensations, greater intensities of the majority of components of the deqi sensation (fullness, spreading soreness, numbness, and electric sensation), and greater spreading of deqi than those who received only superficial needling with thin needles and no manipulation. These data agree with prior studies that investigated the effects of stimulus intensity on the deqi sensation. For example, Benham et al. found that both deep needling and deep needling with bidirectional rotation achieved higher VAS scores for the intensity of the overall needle insertion sensation than did superficial needling with mock deep insertion. Moreover, the scores for the total component sensation and the throbbing sensation, specifically, during deep needling with bidirectional rotation, were higher than those for superficial needling with mock deep insertion [59].

To investigate the specific role of needling depth and sensation, Park et al. used an ultrasound approach. These authors measured needling depth and the frequency of sensations experienced when needling to specific tissue levels (TLs). They found that the frequencies with which participants reported experiencing pricking and sharp sensations were significantly greater with shallower TLs than with deeper TLs. In contrast, the frequencies of deep, dull, heavy, spreading, and electric shock sensations were significantly higher with deeper TLs than with shallower TLs [60]. Researchers similarly reported that deqi intensity was significantly higher when needling occurred with manipulation than without [61, 62] and that needling depth and manipulation were important for maintaining the elevated needle sensation that is associated with needling [63, 64].

Different needling interventions may also influence the sensation of spreading. Our work finds that the distance of spreading in patients who received active stimulation and self-reported deqi was greater than in those who underwent only minimal levels of acupuncture and who self-reported deqi. Jung et al. used sensation mapping to investigate the distribution of acupuncture sensations throughout the body. They found that sensation maps presented with similar spatial configurations in areas close to the stimulated sites in both acupuncture and tactile stimulation. However, sensations in areas distant from the stimulation sites were observed for acupuncture but not for tactile stimulation. Based on this, Jung et al. further concluded that acupuncture stimulation initiated more of a deqi sensation than did tactile stimulation, leading to the spread of the sensation to areas distant from the stimulation site [65].

Notably, the deqi sensation itself cannot be induced directly by the acupuncturist alone; the sensation is somewhat uncontrollable. In the present study, despite using minimal stimulation to avoid deqi, 59.5% of patients in the non-deqi group self-reported deqi sensations. There are several possible reasons for this finding. For instance, this phenomenon may be related to acupoint sensitisation. One study reported that related acupoints were more sensitive in patients with pathological conditions [66]. Given that SP6 is associated with primary dysmenorrhea, when dysmenorrhea occurs, changes in skin resistance and skin temperature may be a manifestation of acupoint sensitisation [67, 68]. This acupoint sensitisation renders deqi more likely. Second, the volunteers who participated in our research had no background knowledge of acupuncture. As such, these patients may have been more sensitive to an experience of deqi sensation.

### 4.3. Limitations

Despite our significant findings, there are several limitations of the present study which warrant some discussion. First, there were unequal numbers of cases in the deqi and non-deqi groups. This design was due to difficulty in achieving a true non-deqi group when using invasive needling at acupoints. Specifically, studies have shown that even non-invasive sham acupuncture can induce deqi sensations similar to those induced by true acupuncture [69–72]. Thus, as per recommendations made in our prior report, we enrolled patients in the deqi and non-deqi groups at a ratio of 1:3 [29]. In so doing, we ensured that the number of patients who did not report deqi in the non-deqi group was similar to the number of patients who self-reported deqi in the deqi group. Second, aspects of this study are subjective. Namely, we asked patients to self-report their sensory experiences. Previously, we investigated the feasibility of using objective measures, such as acupoint surface temperature and evoked somatosensory potential, to evaluate deqi. These methods require further optimisation before they can be used in broad research contexts, however. Third, deqi is reliant on both the perception of the acupuncturist and the experience of the patient [73, 74], yet our study only analysed the patient reaction. Fourth, although there were no significant differences in the baseline characteristics of the two groups, the duration of disease history (from 6 months to 15 years) was quite long. Lastly, the sample size used in the present study was relatively small. Further studies using both subjective and objective evaluation methods to investigate deqi with larger sample sizes and considering the mechanisms underlying the phenomena we report here should be conducted. In addition, other types of pain/pathology might be extended to investigate the influence of deqi on the treatment effects and the underlying mechanisms of it.

## 5. Conclusions

In the treatment of PD patients with a* cold and dampness stagnation* pattern by SP6 needling, a deqi response of moderate intensity is more likely to facilitate prolonged analgesic effects than a robust intensity one. Future research with larger sample sizes using more objective indexes is required to further investigate the relationship between deqi and the effects of acupuncture treatment in different diseases.

## Figures and Tables

**Table 1:** Baseline characteristics of patients (Mean ± SE).

Demographic information	Deqi group (n=15)	Non-deqi group (n=49)	p
Age (year)	21.40 ± 0.65	21.55 ± 0.43	0.974
Onset age (year)	15.13 ± 0.49	16.22 ± 0.35	0.079
Period (day)	5.80 ± 0.30	5.39 ± 0.14	0.219
Menstrual cycle (day)	29.87 ± 0.85	30.22 ± 0.52	0.619
Disease duration (month)	79.53 ± 9.36	64.08 ± 6.34	0.537
VAS-P value (mm)	52.80 ± 2.95	54.90 ± 1.43	0.362

Note. VAS-P: VAS for pain.

**Table 2:** Pain reduction at different time (Mean ± SE).

	Subgroup A	Subgroup C	Subgroup A+C
	(N=14)	(N=25)	(N=39)
VAS-P_0_ − VAS-P_30_	23.79 ± 5.21	17.20 ± 2.26	19.56 ± 2.38
VAS-P_0_ − VAS-P_40_	30.21 ± 4.62	23.04 ± 2.48	25.62 ± 2.33
VAS-P_0_ − VAS-P_50_	32.93 ± 4.64	28.00 ± 2.98	29.77 ± 2.52
VAS-P_0_ − VAS-P_60_	40.36 ± 3.78	32.36 ± 3.61	35.23 ± 2.72

Note. Subgroup A: patients in the deqi group who experienced deqi. Subgroup C: patients in the non-deqi group who experienced deqi. Subgroup A+C: patients from subgroups A and C who experienced deqi. VAS-P_0_-_60_: VAS for pain before treatment, at needle removal, and 10, 20, and 30 min after needle removal (indicated by subscript).

**Table 3:** Correlations between the intensity of deqi sensation and the effect.

	Subgroup A		Subgroup C		Subgroup A+C	
	(N=14)		(N=25)		(N=39)	
	r	p	r	p	r	p
VAS-P_0_ − VAS-P_30_	-0.554^*∗*^	**0.040**	0.21	0.313	0.129	0.434
VAS-P_0_ − VAS-P_40_	-0.381	0.179	0.286	0.166	0.196	0.233
VAS-P_0_ − VAS-P_50_	-0.375	0.186	0.422^*∗*^	**0.036**	0.229	0.161
VAS-P_0_ − VAS-P_60_	-0.328	0.252	0.283	0.171	0.255	0.117

Note. Subgroup A: patients in the deqi group who in fact experienced deqi. Subgroup C: patients in the non-deqi group who experienced deqi. Subgroup A+C: patients who experienced deqi in groups A and C, combined. VAS-P_0_-_60_: VAS for pain before treatment, at needle removal, and 10, 20, and 30 min after needle removal (indicated by subscript). *∗* means p ≤ 0.05.

## Data Availability

All data are fully available without restriction.
